# Book Reviews

**Published:** 1819-08

**Authors:** 


### CRITICAL ANALYSIS

OF

### RECENT PUBLICATIONS, IN THE DIFFERENT BRANCHES OF MEDICINE AND SURGERY.

"I would have men know, that, though I reprehend the easie passing over of the causes of things, "by ascribing them to secret and hidden vertues and properties; (for this hath arrested and laid "asleepe all true enquiry and indications;) yet I doe not understand but that, in the practical "part of knowledge, much will be left to experience and probation, whereunto indication cannot "so fully reach: and this not only in *specie*, but in *individuo*. Yet it was well said, *Vere scire*, "*esse per causas scire*."—BACON.

*The Dublin Hospital Reports, and Communications in Medicine and Surgery*. Vol. II. 8vo. pp. 389. Hodges and M'Arthur, Dublin; and Longman and Co. London. 1818.

THIS volume has already been introduced to our readers in the Sketch of the History of Medicine, given in the last Number of this Journal. We now proceed to fulfil the promise then made by our colleague, that a particular review of it would be given on a future occasion. It commences with

*A Report of the Hardwicke Fever-Hospital, for the Year ending on the* 31*st of March*, 1818; *including a brief Account of an Epidemic Fever in Dublin*. By JOHN CHEYNE, M.D. F.R.S.E. M.R.I.A. &C.

THIS report is to be viewed as a continuation of that given by Dr. CHEYNE in the first volume, and is occupied chiefly by observations relative to the type of fever described as the third species in the former report, and which became the epidemic of summer, and was generally prevalent over Ireland.

In order to form a correct estimate of the value of this report, it should be regarded in its relations to the observations previously made by the same author, as well as to the character of the existing season, and various local circumstances which cannot be embraced in an analytical review: it is only those parts of it which bear a more general relation to the nature of fever, and its appropriate mode of treatment, that can form the subject of our remarks; and, from the full account given on a former occasion of Dr. Cheyne's practical conclusions from an analogous form of disease, we need now only notice those circumstances which possess the most evident novelty, or which, from their importance, can hardly be too often impressed on the mind of the medical practitioner.

In many patients, Dr. Cheyne thought the fever appeared to arise from contagion; at least, it was not easy to account for the number of individuals belonging to the same family, who were brought to the hospital about the same time, or in immediate succession.

"Some patients felt an unaccountable dejection of spirits for several days before seizure; some continued at work or labour for several days after their illness began in the shape of a head-ach, which frequently intermitted; in a few, the disease began with intense head-ach without rigor, the patients being, as they said, at once knocked down; some referred their illness to catarrh or indigestion, and had no suspicion, for the first three or four days, that they laboured under a fever; but, generally speaking, there was nothing unusual in the commencement of the disease. There were rigors, confined bowels, anorexia, head-ach, sometimes vertigo, dryness of the nostrils, severe pains in the loins and back, and debility; and these symptoms were soon followed by increase of temperature, a degree of fullness of the features, and flushing of the face; which last was an universal symptom with all who were admitted within the first week or ten days of their illness. The tongue, in the beginning of the fever, was generally white or grey, slightly swoln, with florid edges."

Symptoms of pulmonic irritation usually set in early. During the months of April, May, and June, of 175 patients, at least three-fourths had cough, with pains or oppression of the chest, and quickened respiration. In many of these there was expectoration of bloody mucus.

In the more severe cases, a state of delirium appeared about the end of the first week, which degenerated into sopor, often accompanied with subsultus tendinum. About the period at which delirium set in, the tongue had often a dark-yellow or brown stripe in the middle, the edges being clean and thinly covered with white mucus. As the soporous state went off, the blackness and dryness of the tongue went off also, leaving that organ in a more natural condition, more expanded, and again white with florid edges, and moist from a return of secretion, which was sometimes copious.

The pulse, in general, was more or less frequent, according to the severity or mildness of the disease; but there were several alarming cases in which it never exceeded eighty.

When delirium set in, the symptoms of pulmonic irritation often abated, and the head-ach also; and, when reason was restored, in some few patients, the pulmonic affection recurred.

The temperature of the body was in general very high, frequently from 104° to 109° Fah. This phenomenon accompanied the fever when epigastric tenderness was the most urgent local affection, as frequently as when affection of the lungs was the predominant symptom of the early period of the fever. The circulation was generally, but not always, proportionably quickened; and the respiration was even less affected during the excess of temperature than the circulation. In forty patients, in whom the temperature exceeded 104°, there was only one death; and in the majority of fatal cases, the temperature did not exceed 100°.

"Venesection (Dr. Cheyne remarks) sometimes lowered the temperature; frequently it produced no change; and in several instances the thermometer rose two or three degrees after blood-letting, even when that measure greatly relieved the patient. It is clear that venesection was not contra-indicated by excess of temperature alone; since, in nineteen patients of a temperature which raised the thermometer above 104°, in whom blood-letting was practised, there was no instance of death.

"In examining the disordered state of the vital functions during the summer of 1817, with a view to the prognostics of continued fever, we derived more information from the state of the breathing than from the pulse, and more from the pulse than from the temperature of the body.

"Among such patients as were admitted early, and were treated upon a strictly antiphlogistic plan, there were many instances of crisis on the third or fourth day, the disease appearing as a febricula, or perhaps rather as an extended ephemera; and these specimens of mild fever occurred even among those who came from houses which afforded us instances of the disease in its worst form: the disease, however, was fatal, in a large proportion, among such as came from houses which we supposed were infected, and in these persons relapses were very frequent. On the other hand, in many who denied having had any communication with patients in fever, the disease was attended with severe symptoms, and ran the usual course. In a word, the fevers which we supposed arose from contagion, and those which seemed to originate in intemperance, cold, fatigue, &c. in which we could discover no trace of contagion, were so shaded into each other, that it was impossible, by their symptoms, to demonstrate any difference between them."

We pass over the observations of Dr. Cheyne on the signs of the critical phenomena, as well as some remarks on the appearances on dissection, from their having been already noticed in the Historical Sketch, to the general account of the remedial measures that were adopted.

"During the first ten or twelve days, the treatment was strictly antiphlogistic. In the cases which terminated before the end of the second week, it was generally antiphlogistic throughout. First, the bowels were thoroughly purged; and then, in all the milder cases, the disease was left to cold water or whey, cool air, and sponging the head and neck and chest with vinegar and water, together with a purgative when there was not more than one stool in the day. In the more protracted cases, the cordial plan of treatment gradually took place of the antiphlogistic: provided there was no inflammatory determination, from four to eight ounces of port-wine were allowed daily; from the latter quantity, every advantage which seemed attainable from wine, was procured. About the eleventh or twelfth day, provided the cough was subdued or had become moist, and there was no head-ach or great flushing, and no tension or tenderness of the epigastrium, I generally ordered wine on the patient's complaining of weakness, or on debility being evidenced in the position of the patient, languor of the circulation, or on the appearance of symptoms which indicate irregularity in the supply of the nervous power; as muttering, low delirium, tremors, subsultus, floccitation, &c.; or on the tongue becoming shrivelled, dry, and black. Along with wine the calomel bolus was given, generally every second day.

"There was another condition of the disease, in which a moderate quantity of wine was allowed. When, between the second and third week of the fever, the patient's appearance was nearly natural, save that his complexion was high, his tongue nearly clean, only perhaps too florid; and when, with these symptoms, the heat of the surface was great, and the complaint of weakness considerable: in such a state, wine was often very useful; to which were added, saline diaphoretics, an occasional purgative, and fomentations to the lower extremities.

"In offering a few observations on blood-letting, it is necessary that I should begin by correcting an error into which I have fallen in my first report. I have there said, that, in two or three cases in which venesection was performed during the exacerbatio critica, the salutary effort of the constitution was interrupted, and the fever went on for several days longer: the term exacerbatio critica ought, in strict propriety, to be confined to the struggle which is apparent before the rigor or sweat takes place, in which case venesection is not always injurious; for, in several instances, mistaking the purport of the symptoms which constituted the exacerbatio critica, I ordered the patient to be let blood, and perspiration and perfect crisis followed the operation. Had I been fully aware of the nature of the struggle in these cases, I would have left the disease to its course. Nevertheless, it is certain that blood-letting in the first period of crisis was not in any instance injurious: the bleeding, alluded to in my first report, which interrupted the salutary effort of the constitution, was performed in the second or third period. The effect of blood-letting in the first stage of crisis, may be considered as analogous to that produced by blood-letting in the hot stage of remittent fever; a practice which was common fifty or sixty years ago, to procure a more speedy and complete remission.

"In April, May, June, July, and August, of about 300 patients admitted into No. 1 and No. 4, 149 were let blood; some of these, three or four times. Of these, immediate relief after blood-letting was experienced by 94; but I am convinced that a much greater number were in an improved state on the day after they were bled: yet the blood drawn was not sizy in one case of twenty, if we except the relapses, and those cases in which blood was drawn to relieve the inflammatory affections which were apt to occur during convalescence.

"Bleeding did not appear to me injurious in any one instance in which it was performed in my wards. Of the 94 patients above mentioned, who were let blood with advantage, 69 had symptoms of pulmonic irritation, and almost every one of these had head-ach also; fifteen were without pulmonic disturbance, but had severe head-ach with flushed eyes, and most of them a tendency to delirium; and three had either epigastric tenderness or tormina and tenesmus. Nearly three-fourths of the patients admitted had pneumonic symptoms."

Tenderness of the epigastrium was with more certainty relieved by topical than by general bleeding, although the latter was frequently employed also. Venesection was seldom repeated, but the application of leeches not unfrequently. After the first or second time of applying them, a vesicatory was frequently ordered to the epigastrium, small doses of neutral salts were given, or some of the saline diaphoretics; and the disease then pursued a more temperate course. We shall not repeat Dr. Cheyne's more general remarks on the use of blood-letting, and other remedial measures; but we must transcribe the following paragraph:

"Of purgatives, cooling drinks, cleanliness, including frequent change of linen and personal ablution, large airy apartments, and thorough ventilation, there appears now to be but one opinion among physicians. With regard to blood-letting, mercury, opium, and wine, (to some one of which, in many otherwise excellent works on fever, an undue bias may be discovered,) I beg to remind the young, inexperienced, and ardent practitioner, that they are remedies applicable only to particular cases; and, with respect to the use of such powerful means, it may be observed, that, while the perfection of our art consists in knowing the exact point at which expectation should yield to action, the greatest authorities in medicine have been more apprehensive of the officiousness of zeal, of the *nimia diligentia medici*, than of that degree of distrust in the resources of prescription, which will prevent us from interfering with the operations of nature upon every trifling alarm."

Symptoms of dysentery occurred in some patients, after the beginning of October, and they not unfrequently formed a part of the disease during the whole winter. In dissections, the mucous membrane of the stomach and intestines was oftener in a pulpy and vascular state, and coated with a morbid secretion, than it had been during summer; "but the brain still continued the chief seat of the morbid appearances. I do not recollect a single dissection in which the remains of an excited state of the vessels of the brain did not appear."

"I never (Dr. Cheyne observes) witnessed so large a proportion of patients in fever, jaundiced, as during the summer of 1818. Now, while I am drawing up this report, (July 1818,) we have what would have been counted by some of the older writers, a strongly-marked bilious constitution, which they would probably have referred to the uncommon and long-continued heat and drought of the season. The great majority of these cases probably depend upon congestion and active absorption of the bile. The icteroid colour generally yielded to leeches applied to the right hypochondrium, or cupping and scarifying; sometimes blisters, and a solution of neutral salts, to which a few doses of the blue-pill were generally added; but many of the cases of this affection doubtless admitted of a spontaneous cure."

On some wards of the Whitworth Hospital being appropriated to patients who were suffering the sequelæ of fever, Dr. Cheyne, anxious to study fever in its effects, and thus to complete his view of the epidemic, obtained the charge of these wards. He found the most common sequelæ to be diseases of the mucous and serous membranes; tubercular consumption, called into activity by excitement of the bronchial membrane, and which always ran a rapid course; hydrothorax and hydrocardia, hæmatemesis, dysentery, ascites, and ophthalmia. Chronic rheumatism was not unfrequent; and there were some other affections of more rare occurrence: mania, paralysis, and an affection, not confined to the female sex, resembling phlegmasia dolens.

We shall conclude our extracts from this highly valuable report with the following paragraph, which we are sure will be perused with general regret:

"It was originally my intention (says Dr. Cheyne) to continue my labours in the Fever Hospital for another year, expecting, in a period of three years, to meet with most of the common varieties of the continued fevers of this country; but, by the resignation of my friend, Dr. Edward Percival, a part of the hospitals of the House of Industry, which does not contain any patients in fever, has fallen to my charge; and hence, as I no longer possess the same ample opportunities of observing the phenomena of fever, this shall be the last publication on that subject with which I shall trouble the reader."

*Surgical Report: containing an Account of those Affections of the Penis which are generally considered as primary Symptoms of Syphilis; with the Modes of Treatment employed in the Richmond Surgical Hospital.* By C. H. Todd, Member of the Royal College of Surgeons in Ireland, &c.

This report is said to form the first of an intended series: the present comprises observations on phymosis and paraphymosis; erysipelatous inflammation, and gangrene of the penis; with some general remarks on ulcers of the organs of generation, and a gangrenous affection of those parts occurring in old men. We shall adduce an account of the last-mentioned disease, as the only part of the report that possesses the least novelty. We can only account for its appearance in this volume, and the publication of it by Mr. Todd, by supposing that he intends to give a complete history of the diseases of the organs of generation; and that he has thought proper to comprise in it what is generally known, as well as the novelties he may have to adduce. We should also remark, that but few of the diseases here noticed, are "*generally considered as primary symptoms of*

*syphilis.*" The following is the account of the form of disease to which we have above alluded :

"Old and debilitated men are not unfrequently received into the Richmond Surgical Hospital, labouring under a peculiar form of mortification of the penis, which in no instance could I trace to a venereal origin. The species of gangrene of the penis to which I allude, is not preceded by any marked symptoms of inflammation; at least, if inflammation does exist at any period, it is in a degree so slight as to be generally unnoticed by the patient. The appearance of actual mortification is the first subject of alarm; and, so great is the apathy with which such patients are affected, that even this is unattended to, until a considerable portion of the penis has sphacelated.

"Patients of this description are seldom able to give a satisfactory detail of the history of the case, and they uniformly exhibit an indifference in regard to the origin and consequences of their disease, which is quite unaccountable. As far as I have been able to ascertain, the affection commences with a slight œdematous thickening, accompanied with some degree of soreness of the prepuce: in a few days, a black spot is observed on or near the extremity of it, which gradually spreads without much pain, until the entire penis is destroyed; in some, the gangrene extends to the scrotum and pubes, but most commonly the patient dies before this can occur.

"During the progress of this disease, the patient is extremely low and weak; his countenance pale and cadaverous; his tongue dry and covered with a brown crust; his pulse languid and often irregular, with a cold clammy moisture on the extremities. In some instances, the bowels are inactive; but, towards the latter end, their liquid contents are discharged involuntarily. Patients affected in this way seldom complain of severe pain: on the contrary, I have remarked that they appear very much at ease, and, although they do not sleep except when under the influence of an opiate, will often remain for several hours together without showing a disposition to move or make any exertion; and they are evidently annoyed and irritated by being compelled to take food or drink, or to assist in changing the applications made to the diseased parts. For a few days preceding death, they are incoherent, with a low muttering delirium and subsultus tendinum; then the local disease extends more rapidly, and the sloughs are much more offensive than at the commencement.

"This is clearly a disease of debility, and arises more from a morbid state of the system at large than from any local disease, although it is probable some slight irritation may cause the gangrene to fix on the particular part.

"In the treatment of a patient labouring under this complaint, our exertions must be chiefly employed in restoring and supporting his strength as much as possible: this object is best attained by a liberal allowance of light nutriment and wine, which his attendants should be directed to administer very frequently. I have repeatedly tried the Peruvian bark in these cases, in all its forms, without benefit; and that medicine has so often disagreed with my patients, that I have ceased to prescribe it where wine and nutritious broths can be taken in reasonable quantities.

"From the exhibition of opium in cases of this peculiar form of mortification of the penis, I have witnessed the most favourable results; and I am certain that the lives of several of our hospital patients have been preserved by the efficacy of this medicine alone. After the state of the bowels has been regulated, we generally commence the use of opium, by directing one grain of the extract to be taken every sixth hour; and, according to circumstances, gradually increase the dose during the two or three first days, until the patient takes a grain and a half every third or fourth hour. I have seldom found it necessary to exceed this quantity; but, in extreme cases, and particularly if the patient's bowels were too free, two grains of opium have been administered every third hour, until a slight narcotic effect was induced.

"By the judicious selection of local remedies, and their careful and regular application in this most formidable disease, much good may be effected. Stimulating dressings, and especially those of the terebinthinate class, are decidedly preferable to any other: they ought to be changed at least four times in the day, and always renewed after the patient has passed his urine. If the sloughs and the discharge are offensive, the effervescing or carrot poultice may be used in conjunction with these applications; or the stimulating dressings may be omitted for a day or two, the antiseptic poultices applied, and, after as much of the mortified part has been cut off as the surgeon can remove with safety, the dressings may be again resorted to.

"When gangrene has ceased to extend, and the sphacelated parts are separating from the sound, the use of opium should be gradually discontinued: however, the patient must still be supported with a generous diet, and the state of his bowels very carefully attended to; and, as this disease has been known in many instances to recur, we must continue on our guard until the part is completely healed, and the strength of the patient established."

We now arrive at the second part of this work, comprising "Miscellaneous Communications on Medical and Surgical Diseases:" this commences with

*A Case of Obliterated Aorta*, by THOMAS GOODISSON, M.D.; *with some additional Observations*, by PHILIP CRAMPTON, M.D. F.R.S. Surgeon-General to the Army and Forces in Ireland.

We refer the reader for an account of this case, to the abstract given of it by our colleague in his "Historical Sketch." The following are the remarks of Dr. Crampton:

"I have, in the first place, to bear testimony to the general accuracy of Dr. Goodisson's description, which I have very carefully compared with the preparation to which it refers. Those anatomists, who have had the advantage of examining aneurisms which have undergone a spontaneous cure, will have no hesitation in referring the obliteration of the aorta, in Dr. G.'s case, to the effects of such a process. 'The firm fleshy substance, having the appearance of the muscular fibres of the heart, and which filled the aorta for the space of about two inches, which was prolonged upwards beyond the bony sheath, and adhered firmly to the coat of the artery,' very well describes the condensed fibrin which lines the walls of all old aneurisms, and ultimately fills up their cavities when the process of a spontaneous cure has been completed; but, by cutting longitudinally through the diseased portion of the artery, and turning out the condensed coagulum with which it was filled, I was enabled to ascertain the real nature of the changes which the vessel had undergone previous to its obliteration.

"The internal coat, covered with steatomatous and earthy concretions, completely lined the cavity of the dilated portion of the artery: the dilatation itself consisted of three irregular pouches, which proceeded from the anterior and lateral surface of the vessel. It is obvious, therefore, that the disease commenced with dilatation of the artery, in consequence of a previously diseased and weakened state of its coats. That the coats had suffered neither ulceration nor rupture was evident, since, when the coagulum was detached, the internal membrane was found smooth and unbroken, and its surface presented precisely the same diseased appearances which were found on the internal coat of the aorta immediately above and below the dilatation. What the circumstance was which determined the blood to coagulate in so small a sac, (if such the dilated artery can be called,) and so much within the influence of a great current, it is difficult to conceive; but the fact is certain, and must, whether in a physiological or a practical point of view, be considered as one of the greatest importance."*

### *A Case of Femoral Aneurism cured by Tying the external Iliac Artery.* By SAMUEL WILMOT, M.D. &C. &C.

A case in which the operation indicated in the title was performed with success, without the occurrence of any remarkable circumstances.

### *A Case of Apoplexy, in which the fleshy Part of the Heart was converted into Fat.* By J. CHEYNE, M.D. &C.

The very interesting nature of this case induces us to transcribe Dr. Cheyne's history of the phenomena observed before death: the appearances on dissection have been already related.

"A. B. 60 years of age, of a sanguine temperament, circular chest, and full habit of body, for years had lived a very sedentary life; while he indulged habitually in the luxuries of the table.

"This gentleman having had several attacks of the gout in his feet, began a course of magnesia in the year 1813; after which he had only one regular attack of the gout. For many years he had been subject

* Mr. Hodgson observes, in his valuable work "on Diseases of the Arteries, &c." that laminated coagulum is almost universally found in aneurisms in which the coats of the arteries have *given way*; but, in those sacs which consist either in a general or partial *dilatation*, he had never met with it." p. 82.

to severe attacks of catarrh, which ended without much expectoration. He had long been subject to œdema of the ankles in the evening; for two or three years before his death (the time could not be ascertained) he had remarked an occasional intermission in the pulse of his heart.

"In the latter end of January, 1816, he consulted me for a pain in his right side under the false ribs, for which he took calomel at bed-time and salts in the morning, repeating these once or twice; but he neglected my directions with regard to diet: nay, his appetite being remarkably keen, he ate more than usual, and took at least a pint of port wine or madeira daily, as was his habit, and this notwithstanding a hard, frequent cough, which came on after I was consulted by him.

"On the 3d of February, he had walked a good many miles, and came home exhausted, with a fluttering or palpitation of his heart, (for he could not well say which,) in a degree he had not felt before. He ate as usual, and drank six or seven glasses of wine, which he thought relieved the fluttering. He was sitting at tea about nine o'clock, when he was attacked with a severe fit of coughing, during which he fell from his chair insensible. I saw him in three or four minutes after his fall, and found him with a contusion on the upper and left side of the frontal bone; he was confused, and unable to recollect himself; he was conscious that some accident had befallen him, the exact nature of which he declared himself incapable of understanding. His pulse was extremely irregular and unequal. It bounded quickly for several pulsations; then it paused, and went on more quickly, but with less force. He was pale, but none of the muscles were affected with palsy. I lost no time in having blood drawn from his arm, to the amount of nearly a pound. He gradually became more collected, but his pulse continued irregular and unequal; his countenance became flushed; the cough occurred in suffocative fits; and he complained of pain on either side of the tuberosity of the occipital bone. Twelve ounces more of blood were drawn about an hour after the first blood-letting; after which the pulse, though it continued equally irregular, was much softer. He complained of the contusion, and of considerable pain behind his ears. He was removed to bed, the heat of the extremities was restored, and fifteen leeches were applied over the contusion; and he took two pills, consisting of two grains of James's powder, three of calomel, and four of compound extract of colocynth.

"On the 4th of February he had several large bilious stools; his understanding was unimpaired, his recollection restored, and he seemed to comprehend the nature of his illness; and he had a sense of fullness in his head, which led me to order him to lose a few more ounces of blood. It would be tedious and unprofitable to particularize the medicines which were ordered from day to day for this patient: they consisted of a mild mercurial every second or third day, and squills with ammoniacum, &c. These were indicated by the loaded tongue, scanty, high-coloured urine, and dry cough. The expectoration being restored, the squills were laid aside on the 15th of February, as they produced nausea and extreme depression of spirits; and bitter infusion, with tincture of cardamoms and soda, was prescribed. On the 19th, a horse-radish bath was ordered, in consequence of some slight demonstration of gout. On the 21st, he had some smart pain, with slight inflammation in the ball of the left great toe. About this period he submitted with so much dissatisfaction to a reduced diet, and declared himself so much better after food, that we were induced to allow him a couple of glasses of wine, and to encourage him to take carriage-exercise. The irregularity in his pulse never ceased. On the 1st of March, he had a return of the suffocative cough and flushing, with some wheezing, which again seemed to demand blood-letting; which was practised with immediate relief. At this period a blister was applied over the region of the heart, which had become the seat of considerable increase of pain, and a discharge was maintained from the blistered surface, by means of ointment of savine and cantharides: about the 4th of March, the sputa became free and concocted. His tongue at this period was for many days furred and of a dark-brown colour, as if it had been sprinkled with ground coffee; it was expanded, and its edge was moist. On the 25th of March, he began to complain of wheezing, more particularly after exertion, but it sometimes attacked him when he was at perfect rest; his legs and ankles became œdematous, the urine very scanty, much loaded, but without being coagulable by heat. At no period of his illness did his pulse beat more than twelve or fifteen strokes in regular succession. Various diuretics were given; the digitalis was proposed, but he refused to take it. Crystals of tartar, the extractum lactucæ virosæ, nitrous æther, &c. were tried without any benefit.

"The symptoms of dropsy rapidly increasing, on the 9th of April he took a draught of infusion of senna, tincture of jalap, and Rochelle salts, which operated largely. On the 10th of April, he was found in bed, flushed, speechless, and hemiplegiac. How long he had been in that state could not be ascertained, as he had peremptorily ordered his servant not to remain in the chamber with him, and not to come to him in the morning till called. All attempts to relieve him were unavailing; his right side continued powerless, and his attempts to articulate were vain. The only peculiarity in the last period of his illness, which lasted eight or nine days, was in the state of the respiration. For several days his breathing was irregular; it would entirely cease for a quarter of a minute, then it would become perceptible, though very low; then by degrees it became heaving and quick, and then it would gradually cease again: this revolution in the state of his breathing occupied about a minute, during which there were about thirty acts of respiration."*

Dr. Cheyne refers to the Edinburgh Medical Journal (Jan. 1816), for a dissection illustrative of this change of structure.

We believe there is no other similar instance on record; but Kerkring, Bonnet, and Morgagni, give histories of cases

* "The same description of breathing was observed by me in a relative of the subject of this case, who also died of a disease of the heart; the exact nature of which, however, I am ignorant of, not having been permitted to examine the body after death."

where the heart was covered with a coat of fat of great depth, with but little appearance of muscular structure in that organ. It is worthy of remark, that, in the case related by Morgagni, the patient also died of apoplexy. VICQ-D'AZYR describes with much accuracy a similar change in other parts of the muscular system.

*A Case in which Suffocation was produced by a Portion of solid Food in the Œsophagus.* By JOHN KIRBY, A.B. Member of the Royal College of Surgeons in Ireland, Senior Surgeon to St. Peter's Hospital, and Lecturer on Anatomy and Surgery at the Anatomical Theatre, in Peter-street, Dublin.

The only useful indication furnished by this case, as Mr. Kirby observes, is, that it tended to confirm the opinion that it is not on the mechanical obstruction of the trachea that the cause of death immediately depends, when a solid substance is arrested in its descent through the upper part of the œsophagus, so much as on the spasmodic constriction of the muscles of the glottis.

*An Account of an Endemic Disease of Ceylon, entitled Berri Berri.* By J. RIDLEY, Esq. Surgeon in the Royal Regiment of Artillery.

This is a comprehensive, and apparently very accurate, history of the disease above designated; but it is too partially interesting to detain us on the present occasion: besides which, it had been previously well described by Dr. CHRISTIE, in an "Essay on the Diseases incident to Indian Seamen, or Lascars, on long Voyages; by W. HUNTER, A.M."

*An Account of the Endemic Fever of Spain, as it occurred at Carthagena in the Autumn of* 1812. By THOMAS PROUDFOOT, then Assistant-Surgeon of the 27th Regiment.

This paper contains some observations and judicious remarks on an epidemic disease, which appears to have been what is termed yellow-fever; but they are not of sufficient novelty, after the detailed and accurate histories we now possess of that disease, to lead us to occupy the pages of our Journal with a detailed account of them; and a general view of Mr. Proudfoot's pathological remarks was given in the last Number of this work.

[*To be continued.*]

*On the Mimoses; or a Descriptive, Diagnostic, and Practical Essay, on the Affections usually denominated Dyspeptic, Hypochondriac, Bilious, Nervous, Chlorotic, Hysteric, Spasmodic, &c.* By MARSHALL HALL, M.D. Author of a Treatise on Diagnosis; formerly Senior President of the Royal Medical Society, and Physician's Assistant in the Royal Infirmary, Edinburgh. 8vo. pp. 176. Longman and Co. 1818.

A PROMISE was made, some time since, when a partial view of this work was given in our Journal, that a particular account of it should be adduced at a future period; and it is in conformity with that promise, that we take it up on the present occasion.

It should be well understood, that the proper object of a critical review is to show, in the first place, the character of a work, and the value of its doctrines when generally and particularly considered: this, in justice to authors and the interests of science, should form the first and greatest care of the critic; he may then consult the propriety and utility of his own production, and, for the exertions he has made to display the merits of an author, be permitted to adduce such extracts from the subject of his labours as will enrich his own work, whilst they serve to illustrate or show the correctness of his critical observations and reflections. Now, there are some literary productions to which it is difficult to apply these principles; works, of which every part is so connected with the whole, that any insulated portion of them would have an incompleteness of character and apparent object, and which, being themselves condensed in manner, do not admit of an abstract being formed of their contents. This is the case with the present treatise by Dr. Hall. Unless this work be perused throughout, and with rather a particular degree of attention, its objects will not be evident. On a partial or superficial view, it may, indeed, appear to the reader to be without a precise object; and this idea will probably be favoured by its title, which is certainly not happily chosen. But, if it be perused with assiduity, and duly reflected on, it will be apparent that it is calculated to be of great utility to the medical practitioner; chiefly as a guide to the diagnosis of a very obscure and severe class of diseases. Although concise in form, and unostentatious in its character, it comprises the results of long and extensive experience, and evidently of much assiduous reflection. To those who have not perused Dr. Hall's "Treatise on Diagnosis," we observe, that this physician has an express talent for semiological observation, and has successfully devoted much of his useful labours to the promotion of this important, though generally too much neglected, part of medical knowledge.

In addition, then, to the brief account previously given of Dr. Hall's observations on the mimoses, we shall only adduce the introductory part of this work, to show its objects; and the concluding paragraphs, which contain as extensive an account as we can give, as an exposition of its doctrines; and, being presented in the words of the author, they will appear in the most favourable manner, as relates to precision and perspicuity.

"There is a class of disorders, each of which is singularly characterized by being complex, multiform, various, and changeable, and by imitating, from the appearance and predominance of particular symptoms in particular instances, other diseases very different in their nature.

"These affections have been variously, and perhaps too exclusively, attributed, by some authors, to a state of derangement in one or more of the chylopoetic viscera; and by others, to an unequal and undue distribution of the blood, by which a state of arterial excitement or of venous congestion, is induced in some particular organ, or in some particular part of the sanguiferous system. I have scarcely ventured, in this work, to enter into any speculation relative to the pathology of the affections of which it treats; for this part of medicine, notwithstanding the ingenuity of some late theorists, seems scarcely to have advanced from the state of conjecture and uncertainty described by Celsus, whose words* are still, in every sense, but too admissible. My object has rather been to present the reader with what a cautious and patient observation has taught me respecting the history, the causes, description, diagnosis, and treatment, of these disorders.

And, as the real nature and connexion of the general and topical affections in these complaints may frequently be dubious, I have deemed it advisable to appropriate some new term, which might, without implying any opinion on this subject, sufficiently express a prominent and important feature of this class of morbid affections. The denomination MIMOSIS, from the Greek word μιμος, *imitator,* will at once denote a remarkable peculiarity of these disorders, and serve to impress the mind with the necessity of distinguishing, in local affections, between those which belong to the present class, and others which are either primary or have a different origin.

"For a similar reason, I have discarded the terms bilious, nervous, spasmodic, &c. as denominations for diseases; and have reserved them only to denote certain symptoms of morbid affections. In the latter sense, their import is generally understood and sufficiently definite;

* Cum hæc per multa volumina, perque magnæ contentionis disputationes, a medicis sæpe tractata sint atque tractentur; subjiciendum est, quæ proxima vero videri possint. Ea neque addicta alterutri opinioni sunt, neque ab utraque nimium abhorrentia; media quodammodo inter diversas sententias: quod in plurimis contentionibus deprehendere licet, sine ambitione verum scrutantibus, ut in hac ipsa re. Nam quæ demum causæ, vel secundam valetudinem præstent, vel morbos excitent, ne sapientiæ quidem professores scientia comprehendunt, sed conjectura persequuntur. Cujus autem rei non est certa notitia, ejus opinio certum reperire remedium non potest. Verumque est, ad ipsam curandi rationem nihil plus conferre, quam experientiam.—CELSI PRÆF.

but, in the former, they could only serve to satisfy the mind with vague conceptions of the affection, and to check the investigation of its particular and individual nature.*

"It was originally intended to publish the following essay in a larger form, accompanied by representations of the complexion, tongue, tinge of surface, and of the hands. It is now found necessary, however, to leave the task of procuring plates to some one more fortunately situated, or at least to a subsequent period. In the mean time the text, it is hoped, will be found a faithful portrait from nature, not unacceptable to the reader of practical medicine."

The following constitute the concluding observations of the author;

"Too much praise cannot be conferred on those members of the profession, who have so well elucidated the nature and treatment of some of the subjects of the preceding pages. There is no doubt, indeed, that this investigation of the mimoses was suggested to me by what I have learnt from the invaluable labours of Dr. Hamilton, Mr. Abernethy, and other respectable writers. My situation in Nottingham, however, has been a principal cause of fixing my attention on a class of disorders, of which the usual causes are sedentariness and confinement. This town, so celebrated for its manufactories of cotton stockings and lace, embraces a very extensive population, a great majority of which (men, women, and children) are engaged from morning till evening in the numerous sedentary occupations which these manufactories imply, deprived of the salutary influence of pure air and gentle exercise.

"To these numbers, which are peculiar to my situation, must be added the sedentary amongst the remaining part of the population,—the literary, persons of a delicate mode of life, females in general, tailors, mantua-makers, and the youthful inhabitants of the schools.

"Nor is sedentariness the only cause of the mimoses, the operation of which is peculiarly frequent amongst the poor of Nottingham. I have noticed the frequent occurrence of the mimosis decolor in cooks and housemaids. The same remark applies equally to those persons who are much engaged in 'ironing,' and, of consequence, much confined to an atmosphere over-heated by stoves for the purpose of quickly drying the articles subjected to this process.

"To this view of the causes of the mimoses, peculiar to a manufacturing town, may be added the baneful influence of a confined and impure atmosphere, and an indigestible and poor diet; an influence which we learn to estimate more perfectly, by adverting to the impediment they afford to our attempts to cure, and to the beneficial effects of the country-air and exercise, with a proper and nutritious diet.

"A certain activity of the body would appear to be necessary to insure the peristaltic movements of the intestines, and, in consequence, the propulsion of their contents. During sedentariness, these movements are probably retarded, the alvine evacuation becomes more scanty or less frequent, and the intestines remain loaded.

* See the author's *Treatise on Diagnosis*, Part i. pp. x. 2, 3.

"From this loaded state of the bowels, their functions, and those of all the chylopoetic viscera, most probably become deranged. The alvine contents become disordered merely by delay; and their presence induces in its turn a disordered state of the functions, secretions, or actions, of all the organs contributory to digestion; and at length of other organs more remotely situated in the animal frame.

"The functions of the parts within the mouth become first obviously disordered. The secretions become morbid; the tongue becomes loaded and swollen; the gums red and tumid; the breath tainted; and the saliva sometimes profuse and offensive. The complexion and the skin become morbid, and there are the appearances observed in the mimosis acuta, or the mimosis decolor. This condition of the complexion and skin varies with the state of the original disorder, and with that of the tongue and internal mouth, of which it affords indeed an index. With the state of the mouth and skin, that of the secretions and other functions of the whole course of the alimentary canal and the contributory digestive organs, the liver, the pancreas, &c. may be presumed to be all morbidly affected. Digestion is variously deranged; the contents of the bowels become unnatural; and thus reciprocally. According to the state of things, nutrition is impaired, or the sensations are uneasy and painful. To term these disorders stomachic, intestinal, hepatic, or bilious, would alike afford partial and inadequate views of this comprehensive subject. As co-existent or subsequent links of this chain of sympathies, the functions of the brain, heart, respiration, stomach, intestines, uterus, bladder, &c. become variously affected. The muscular system and the senses also suffer in different instances; and nutrition, absorption, and secretion, are impeded or impaired.

"From this view of the subject, the character of the mimoses may be deduced. And the recurrence of this word leads me once more to apologize for the introduction of a new denomination for these diseases. I have been induced to adopt this term, first, to prevent a great deal of circumlocution; and, secondly, because I could find no other in use, which was not objectionable from implying some hypothetical view of the subject. These motives, I trust, will appear sufficient to justify the innovation. I can at least conclude in the words of Morgagni, 'Longe mihi potior cura est veritatis quam novitatis.'"

---

*An Essay on the Diseases of the Excreting Parts of the Lachrymal Organs.* By William Mac Kenzie, Member of the Royal College of Surgeons, of the Medical and Chirurgical Society of London, and Lecturer on the Anatomy and Surgery of the Eye. 8vo. pp. 95. London, Anderson and Chase. 1819.

This essay constitutes an addition to our medical literature of considerable value: the subject of it is particularly interesting, and is by no means well understood by the generality of English surgeons. Under the term Fistula lachrymalis, various forms of disease have been comprised, differing in their origin, nature, and in the treatment they require; and, as is ordinarily the case, a want of accuracy and precision in the definition of these maladies, has been attended with a want of due discrimination in the measures employed for their relief. The German surgeons excel, on many occasions, in nice discrimination of the various forms of disease which may occur in particular organs, and especially in those affecting the eye, and parts subservient to it. To supply in part the deficiency of information in our language on this point, is the intention of the present work; and it is an object which Mr. Mac Kenzie is well qualified to effect, both from his literary researches, and his opportunities for personal observation during a residence for some time in Germany, and especially his experience under the tuition of Professor Beer, of Vienna. Yet Mr. Mac Kenzie is not to be considered in this instance merely as an imitator: he has differed, in some respects, in his arrangement of the subjects of the present essay from Professors Beer and Schmidt, whom he chiefly follows; and advances some original observations of practical utility.

The author distinguishes the diseases of the excreting parts of the lachrymal organs into the following varieties: I. *Wounds of the lachrymal canals.* II. *Erysipelatous inflammation of the parts covering the lachrymal sac.* III. *Acute inflammation of the excreting parts of the lachrymal organs.* IV. *Chronic blenorrhœa of the excreting parts of the lachrymal organs.* V. *Stillicidium lachrymarum.* VI. *Fistula of the lachrymal sac.* VII. *Caries of the os unguis.* VIII. *Relaxation of the lachrymal sac.* IX. *Mucocele of the lachrymal sac.* X. *Obstruction of the lachrymal canals.* XI. *Obstruction of the nasal duct.*

This arrangement is not, we consider, wholly free from objections. Fistula of the sac is always a consequence of some of the other forms of disease above-mentioned, and may accompany nearly the whole of them; it must also vary in its character, according to the causes on which it depends, and requires a relative diversity in the measures for its cure: it would, therefore, probably have been more properly treated of in connexion with the diseases which it may accompany, than in an isolated manner. The comprising of caries of the os unguis amongst the diseases of the excreting parts of the lachrymal organs, is not quite correct. We are also not perfectly satisfied with the making a distinct variety of relaxation of the lachrymal sac; but, on turning to the work of Prof. Beer* on Diseases of the Eye, we find that he also considers it in a distinct manner, under the appellation of hernia of the lachrymal sac, which must be

* *Lehre von den Augenkrankheiten, als Leitfaden zu seinen Oeffentlichen Vorlesungen.* Von George J. Beer. Wien. 1817.

considered as high authority in support of its propriety; although Prof. Beer, as well as Mr. Mac Kenzie, describes it to be a consequence of previous excessive distention of the sac from collection of mucus, &c. in that organ, from either acute or chronic inflammation of its lining membrane. A similar objection may be made to the consideration of stillicidium lachrymarum as a variety of disease, regarding it in its connexion with a morbid state of the *excreting* parts of the lachrymal organs. With those exceptions, we consider the arrangements adopted by Mr. Mac Kenzie as systematically proper, and calculated to be of considerable utility in its application in the practice of surgery. A rapid view of the work will render this evident.

We pass over the first section, since it contains no pathological observations of important novelty, nor any therapeutical precepts not indicated by the general principles of surgery.

In the *erysipelatous inflammation of the parts covering the lachrymal sac*, the appearance of the integuments is similar to that produced by erysipelas in other parts of the body. The irritation extending to the papillæ lachrymales, causes a contraction of their orifices, and a collection of the tears in the nasal angle of the eye; and, when the inflammation is more severe, affecting the lachrymal sac, nasal duct, and canals; the papillæ become so contracted, that the puncta are completely closed; "the nostril upon the side affected is dry, and so uncommonly sensible, that the slightest irritation of the schneiderian membrane causes violent sneezing." These accidents generally disappear when the inflammation of the integuments subsides; but, the author continues to observe,

"If the lachrymal canals have participated more than common in the disease, the re-absorption of the tears does not take place immediately upon the inflammation subsiding. On the contrary, a stillicidium lachrymarum continues, when all the other symptoms have disappeared.

"If the inflammation is severe, and extends beneath the integuments, the lachrymal sac, at the commencement of the second stage, becomes completely filled with mucus, which can always be discharged by pressure. At the same time, the erysipelas ends in a real process of suppuration; the subcutaneous cellular substance becomes disorganized in order to make room for the matter of an abscess: this matter collects between the integuments and the orbicularis palpebrarum; sometimes it makes its way between the fibres of that muscle, penetrates the fibrous layer by which the sac is immediately covered, and comes into contact with the anterior part of the sac, which is already distended by the presence of an inordinate quantity of mucus. At last, the integuments give way in one or more points, and the abscess is discharged. The appearance of the parts is now apt to impose upon a superficial observer. There is the tumor of the sac; there is the fistulous opening of the integuments. He probably pronounces the case to be a fistula lachrymalis, and forthwith opens the sac.

"Let us distinguish this case from another to which it bears some resemblance, but with which it must by no means be confounded. It may happen that the purulent matter accumulated under the integuments has actually penetrated the anterior side of the sac, which thus comes to be filled with pus received from without, in the production of which its lining, or mucous membrane, has had no share. This latter case, which, for the sake of distinction, may be called spurious fistula of the lachrymal sac, must be carefully distinguished both from the former, in which the sac is entire though distended with mucus, and from those diseases hereafter to be described, in which the purulent matter that fills the sac is the result of inflammation of the lining membrane of the sac itself."

We have indulged in the foregoing extensive transcription, because it furnishes a good specimen of the author's accuracy of observance, and will show the importance of precise pathological knowledge in the treatment of the disease.

Some observations on the causes, prognosis, and the general and local treatment, follow; from which we adduce an extract, that, with the preceding account, will convey rather a comprehensive view of the most important part of this section of the work.

"At the commencement of the suppurative stage, the patient must first of all be placed in a warm and dry atmosphere. A dry linen compress ought to be laid upon the affected integuments. Gentle diaphoretics ought to be given. If the symptoms indicate the formation of a subcutaneous abscess, a warm poultice of bread and milk ought to be applied. Still, we must not leave the matter of the abscess to make a way for itself through the integuments; but, as soon as even indistinct fluctuation is perceived, we must open the abscess with the lancet, in order to save the lachrymal sac, and prevent the formation of a spurious fistula.

"If we are not called till such a fistula has formed, let us beware of all unnecessary introduction of probes and syringes into the sac. By means of a small syringe, the fistula is to be washed out once a-day with tepid water, mixed with a little of the vinous tincture of opium. A small quantity of lint dipped in the same tincture, is then to be introduced into the abscess, but not pushed so deep as to enter the lachrymal sac. After the fistula has healed, the blenorrhœa which may remain is to be treated as shall be explained in the fourth chapter."

In the form of disease next considered, *acute inflammation of the excreting parts of the lachrymal organs*, the primary seat of the malady is the mucous membrane of the whole of the excretory parts of the lachrymal organs; this is accompanied with

"Obtuse, deep-seated pain, extending to the nóse, and even to the eye-ball; a swelling appears in the situation of the lachrymal sac, having the shape of a bean, accurately circumscribed, hard, very sensible to the touch, and affected with stinging pain whenever it is pressed. This swelling becomes gradually red, at last extremely red, and then the least touch is insupportable. The papillæ are shrunk, the puncta are scarcely visible; the absorption and conveyance of the tears into the lachrymal sac, and through the nasal duct into the nose, are completely stopped, and a stillicidium, or discharge of tears and mucus, takes place from the nasal angle of the eye. The nostril on the affected side is at first uncommonly moist, but it soon becomes dry, the inflammation extending to the mucous membrane of the nose. The inflammation affects the caruncula lachrymalis and the conjunctiva, spreading also to the orbicularis palpebrarum, and to the integuments of the lower eyelid. The redness about the nasal angle of the eye, even with some degree of swelling even to the cheek, gives to the parts, when viewed at a distance, an appearance as if the integuments were attacked by erysipelas; but, on a nearer examination, the peculiar redness, and all the other characteristics of phlegmonous inflammation, are recognized; and, in the midst of the diffused discoloration and tumefaction, the circumscribed swelling of the lachrymal sac is evident, not merely to the touch, but even to the view."

The author traces the progress and consequences of this affection in a very lucid and satisfactory manner: the most frequent results, when the inflammation has been severe, are thickening of the mucous membrane of the whole organ, and obstruction of the canals and nasal duct. The abundant secretion of mucus, being collected, distends the sac, induces inflammation and consequent rupture of its paries, and the matter is evacuated externally through the fibres of the orbicularis palpebrarum muscle and the common integuments: the consequence of this is the fistulous opening, described more particularly and distinctly in the fifth section of this essay. Sometimes, even after this accident, the natural passage of the tears is spontaneously re-established, and the opening in the paries of the sac will heal. The latter occurrence may also happen whilst the nasal duct alone remains impervious; when the patient is obliged, several times a-day, to press upon the sac to evacuate its contents. If the wound be permitted to heal whilst the canals and duct are both obstructed, the natural secretion of mucus going on will distend the sac, and induce the disease described in the ninth section under the term *mucocele*, by Mr. Mac Kenzie; or what is, not so appropriately, called dropsy of the sac by Prof. Beer.

A more mild degree of inflammation, or irritation, of the same parts, constitutes the next variety, *chronic blenorrhœa of the excreting parts of the lachrymal organs.* This is the most common of the diseases described in this essay, and is treated by the author in rather an extensive manner. The various causes, constitutional and local, from which it arises, and the consequent diversity in its character and the treatment required for it, render it impossible to convey much information respecting it in an abstract. A bean-shaped tumor, from distention of the sac, and a discharge of puriform mucus through the puncta, (for the canals are not obstructed,) are the most usual symptoms. Persons of a scrofulous habit are most frequently the subjects of it; and it is usually particularly troublesome, or solely attacks the patient, during cold and wet weather. The more severe consequences of it are fistulæ, from attacks of acute inflammation ending in suppuration ; induration of the organ; and permanent obliteration of its canals. The obstruction of the nasal duct, in the first instance, merely depends on the tumefaction of its mucous membrane.

This affection is ordinarily much mismanaged by practitioners in general, from a want of correct knowledge of its real nature.

*Stillicidium lachrymarum* is properly distinguished from *epiphora;* the former depending on obstruction of the excretory parts of the lachrymal organs, the latter on increased secretion of the tears; and, in the former point of view, cannot with propriety be considered as a form of disease.

In the section on *fistula of the lachrymal sac*, the author describes, in a particular manner, the different varieties of ulcers connected with the paries of that organ, and the appropriate mode of treatment under the various circumstances in which they appear.

From the chapter on *caries of the os unguis*, we must transcribe the following passage; it describes what we believe is frequently too true.

"The idea of the frequency of caries of this bone, which, notwithstanding the testimony of Sharp and Janin, has continued to prevail, appears to be founded chiefly upon the mismanaging treatment of surgeons themselves; and, above all, is to be attributed to their rude examination of the parts with probes and other instruments. A patient presents himself with fistula of the lachrymal sac; the idea of caries starts up in the surgeon's mind, and he forthwith takes a probe in order to examine whether there is caries or not; he penetrates the posterior part of the lachrymal sac, touches the bone with the point of the instrument, which he moves about to this side and to that, in order to make himself sure of what he is seeking for; and at last, distinctly feeling the bone, which he has denuded, he pronounces the os unguis to be carious."

Caries of this bone but rarely happens, except from fistulous ulcers of the sac, syphilis, and scrofula; and is, consequently, not a subject for any important measures of expressly local treatment.

The *relaxation of the lachrymal sac*, is a consequence of its over-distention by collected mucous or puriform matter in the diseases described in the third and fourth sections, which has destroyed its natural contractility; and it becomes distended by its own mucus, often still secreted in preternatural quantity, and the tears collected in it, into a bean-shaped tumor. The integuments covering it are scarcely or not at all discoloured; it is not painful, it yields easily to the pressure of the finger, and never exceeds the size of a common horse-bean. Its contents are discharged by pressure, either by the canals or the nasal duct, according to the direction in which the force is applied.

"The treatment consists in the use of two distinct means, each of which, as may be seen by the testimony of Pellier and others, is, when used alone, apt to fail.*

"The first is the compression of the sac; and here let it be observed, that the present is the only case in which compression of the sac is useful. In any other disease of that part, this practice would produce the most destructive effects. The compression must be carefully applied, constantly continued, and gradually increased. Machines have been invented for this purpose, but they never fulfil with precision all these conditions. We cannot, by such an instrument as Sharp's or Petit's compressorium, the first invention of which we owe to Hieronymus Fabricius, keep up a regular and an increasing pressure: the compressing surface, upon the least occasion, especially during the night, is disarranged; and the patient is hindered from pursuing his business by the presence of such an apparatus. Graduated compresses, then, are to be preferred; over these a firm leather pad, of a proper form, is to be placed; and the whole is to be supported by a narrow roller passing round the head. In this manner, the pressure takes place exactly upon the part which ought to be acted upon; it can be daily increased; the pad cannot, even when the patient is very restless, be shoved aside; nor need such an apparatus prevent him from following his ordinary employment, even out of doors.

"The second part of the treatment consists in the application of some astringent fluid, both to the external surface of the tumor, and to the internal surface of the relaxed sac. A great variety of astringents might be mentioned as proper for this purpose; such as the sulphate of iron or of copper in solution, an infusion of oak-bark, or diluted alcohol. The graduated compresses are to be moistened twice or thrice daily with the astringent fluid which shall have been selected. A small quantity also of the same fluid is to be dropped into the lacus lachrymarum, and left to be absorbed by the puncta, the patient being laid in a horizontal position, and the compresses somewhat relaxed, but not removed.

"Such is the method of curing a disease, which I have shuddered to see treated by incision of the sac, and the passage of various instru-

---

* Pott—*Observations on the Fistula Lachrymalis.* Works. London, 1808. Vol. i. page 252.

Pellier de Quensy, *Cours d'Operations sur la Chirurgie des Yeux.* Paris, 1790. Tome ii. page 207.

ments through the nasal duct; operations which are to be regarded as the more severe in such cases, because they are wholly unnecessary. As for the Anelian injections, the direct injury of the papillæ, and the over-distention and consequent atony of the puncta, so that they can no longer absorb the tears, are only some of the bad consequences of such a practice. Such injections in this particular case would be attended also by a momentary over-filling of the sac, which would be just undoing with the one hand what we were effecting with the other."

In the *mucocele of the lachrymal sac*, the tumor is accompanied with a purplish colour of the integuments, and it often attains the size of a pigeon's egg; it is very firm to the touch, and its contents cannot be evacuated by pressure, because the canals and ducts are both totally obstructed.

"The colour of the integuments in mucocele has led some authors to describe this disease under the name of varix of the lachrymal sac; and the hardness and size of the tumor, added to its colour, have frequently led those charlatans, who were formerly but too often intrusted with the care of the diseases of the eye, to extirpate the lachrymal sac affected with mucocele, under the idea that they were removing a cancerous tumor."

In the appropriate treatment of this important form of disease, the question is not whether the tumor only can be removed, for this can always be effected by laying open the sac, and turning out its contents: the important question is, whether or not the natural passages can be restored.

*Obstruction of the lachrymal canals* and *nasal duct*, are the affections best understood by the English and French surgeons, being those to which their attention has been chiefly directed in their views of the diseases of the organ under consideration; yet the observations of Mr. Mac Kenzie on these subjects will be perused with advantage.

Our observations on this essay have occupied a much greater space than we usually devote to so small a work; but this has necessarily arisen from the very concise manner in which the author has himself treated the different subjects it embraces: he has effected this as closely as is consistent with perspicuity of pathological description and practical utility. This is a circumstance which will contribute much to favour the benefit that will ensue from his labours, by rendering general the possession of this work by practitioners of surgery. This is no unimportant merit, at a time when productions in medical literature are so numerous, and in general so expensive, as they are at the present period.

We hope to see Mr. Mac Kenzie pursue the course he has here commenced: there are many other points in the pathology of the eye, and the parts subservient to it, on which he might adduce some illustrations, both novel and important to English medical literature.

---

*Practical Observations on the Medical Powers of the most celebrated Mineral Waters, and of the various Modes of Bathing. Intended for the Use of Invalids.* By PATRICK MACKENZIE, M.D. Licentiate of the Royal College of Physicians of London, and Assistant Physician to the Fever Institution, &c. 12mo. pp. 151. London, Burgess and Hill. 1819.

ALTHOUGH this work is intended for popular use, we introduce it to our readers; since it is well calculated to supply a deficiency in our literature on the subjects of which it treats, which most medical practitioners must have had occasion to observe. On recommending patients to go to watering and bathing places, it is usual to find them advance many questions respecting the qualities of the various mineral waters, and their regimen during their use; when a work like the present to refer them to, will be found very convenient. These are subjects on which some degree of popular medical information will be really beneficial, and we are much pleased with the way in which it is conveyed in this work; for, though the author has here chosen to confine his views to the objects to which we have alluded, it is evident that he is well qualified to treat the subjects in a more profound and comprehensive manner: what he has advanced is therefore solid, although adapted to the comprehension of general readers.

We may observe, that Dr. Mackenzie has treated of the different mineral waters with a view to their medicinal, rather than their chemical, qualities; although a general account of the latter is also adduced. He divides them into the cold diluent waters, the chief of which are those of Malvern; tepid diluent waters, which are the Matlock, Bristol, and Buxton; diuretic waters, as the Seltzer; cold stimulant waters, the Spa and Pyrmonte; hot stimulant waters, those of Bath; tonic waters, Tunbridge and Hastings; tonic and aperient, the Cheltenham and Scarborough; tonic, aperient, and diuretic, the Vichey and Carlsbad waters; tonic and astringent, the Hartfell, Brighton, and Sandrocke waters; the purgative, the Seidlitz, Epsom, Leamington, and common sea-waters; alterative and detergent, those of Harrowgate and Moffat; and the hot alterative and detergent waters, those of Aix-la-Chapelle and Bareges.

